# Relative contributions of fasting and postprandial glucose increments, glycemic variability, and non‐glycemic factors to HbA1c in individuals with type 1 diabetes

**DOI:** 10.1111/1753-0407.13388

**Published:** 2023-05-04

**Authors:** Yongwen Zhou, Mao Zheng, Hongrong Deng, Xueying Zheng, Sihui Luo, Daizhi Yang, Xiaodong Mai, Wen Xu, Jinhua Yan, Jianping Weng

**Affiliations:** ^1^ Department of Endocrinology, Institute of Endocrine and Metabolic Diseases The First Affiliated Hospital of USTC, Division of Life Sciences and Medicine, Clinical Research Hospital of Chinese Academy of Sciences (Hefei) Hefei Anhui China; ^2^ Department of Endocrinology and Metabolism The Third Affiliated Hospital of Sun Yat‐sen University, Guangdong Provincial Key Laboratory of Diabetology Guangzhou China

**Keywords:** basal hyperglycemia, HbA1c, postprandial glucose, type 1 diabetes mellitus, 基础高血糖, 餐后血糖, HbA_1C_, 1型糖尿病

## Abstract

**Aim:**

Evidence for contribution of basal and postprandial glucose increment, and glycemic variability to glycated hemoglobin (HbA1c) among adults with type 1 diabetes (T1D) is limited. This study aimed to capture glycemic fluctuation patterns and quantify contributions of these factors to HbA1c levels among adults with T1D.

**Methods:**

HbA1c, continuous glucose monitoring (CGM), and diet diaries were collected and pooled from two clinical trials. Available data sets were divided into HbA1c quartiles: group 1 (≤6.7%), group 2 (6.7%–7.3%), group 3 (7.3%–7.8%), and group 4 (≥7.8%). Area under curve above 110 mg/dL (AUC_>110mg/dL_) in 24‐h profile was defined as overall hyperglycemia and stratified with postprandial hyperglycemia (PHG, AUC_>110mg/dL_ in 3‐h period after meals) and basal hyperglycemia (BHG, AUC_>110mg/dL_ in remaining period). Linear regression analysis was used to estimate the proportion of variance in HbA1c explained by BHG, preprandial glucose, PHG, glycemic variability, and non‐glycemic factors (age, body mass index, hemoglobin, and duration).

**Results:**

A total of 169 550 glucose data in 2409 meals recorded from 102 patients (male/female, 34/68) were included. Age and duration were 35.2 ± 12.6 and 8.9 (2.9, 13.0) years, with 51.0% using pumps. Overall, BHG was four times higher than PHG (*p* all <.05) and between‐group comparisons showed BHG exhibited a progressive increase (group 1 vs. 2, 3, 4, *p* = .053, .086, .006) with fasting contribution of 76.1%, 82.6%, 81.5%, and 84.3% from group 1 to 4. The increment was not significant among groups 2, 3, and 4 (*p* > .05). Factors included in analysis explained a total of 74% of the variance in HbA1c, in which BHG accounted for 32.1% of variance whereas PHG accounted for 24.4%. In group with HbA1c >7.3%, BHG accounted for a higher percentage with 33.8% of the variance in HbA1c.

**Conclusions:**

In our study, basal hyperglycemia better predicts overall glycemic control than postprandial hyperglycemia among adults with T1D. The relative contribution of basal hyperglycemia increased gradually with HbA1c increasing and predominant strategy for insulin titration among T1D is different among different levels of glycemic control.

## Graphical abstract

**Highlights**

- Evidence for contribution of basal and postprandial glucose increments and glycemic variability to glycated hemoglobin (HbA1c) among adults with type 1 diabetes (T1D) is limited.
- In this study, after analyzing a total of 169 550 sensor‐derived data in 2409 meals recorded from 102 T1D adults, we found that basal hyperglycemia (BHG) was four times higher than that of postprandial hyperglycemia (PHG). Factors including BHG, preprandial glycemia, PHG, glycemic variability and age, body mass index, hemoprotein, and duration explained a total of 74% of the variance in HbA1c, in which fasting hyperglycemia accounted for 32.1% of variance and postprandial glycemia accounted for 24.4%.
- The relative contribution of fasting hyperglycemia increased gradually with HbA1c increasing.

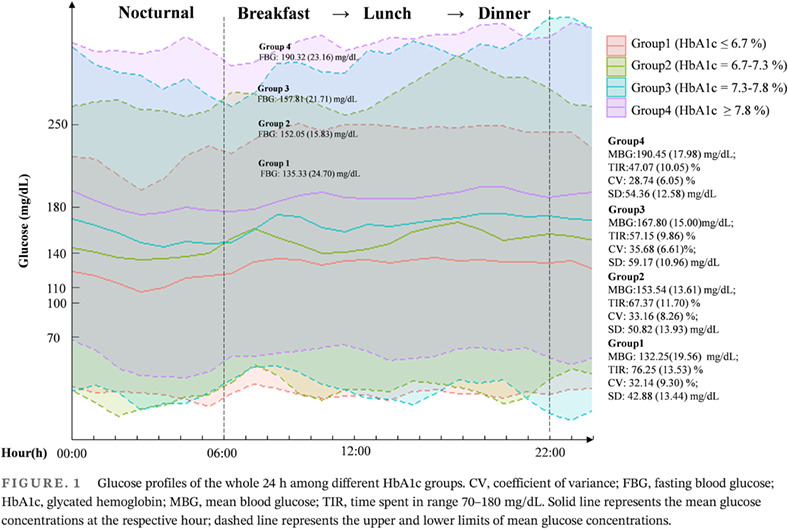

## 摘要

**【摘要】 目的** 目前有关基础和餐后血糖升高以及血糖变异对成人1型糖尿病HbA_1C_水平的贡献的证据有限。本研究旨在捕捉血糖波动模式，并量化这些因素对成人1型糖尿病HbA_1C_水平的贡献。**方法** 收集并汇总了2项临床试验中的HbA_1C_、连续血糖监测(CGM)和饮食日记数据。根据HbA_1C_四分位数将可用数据集分为4组:组1(≤6.7%)、组2(6.7%~7.3%)、组3(7.3%~7.8%)和组4(≥7.8%)。将24小时血糖曲线下面积大于110 mg/dL(AUC>110 mg/dL)定义为总体高血糖，并根据餐后高血糖(PHG，餐后3小时期间AUC>110 mg/dL)和基础高血糖(BHG，剩余时间内AUC>110 mg/dL)进行分层。使用线性回归分析来估计HbA_1c_变异中BHG、餐前血糖、PHG、血糖变异性和非血糖因素(年龄、体重指数、血红蛋白和病程)所解释的方差比例。**结果** 共纳入102例患者(男性/女性，34/68)的169 550条数据和2 409个餐次记录。年龄和病程分别为35.2±12.6岁和8.9(2.9,13.0)年，其中51.0%使用泵。总体而言，BHG高于PHG4倍(*P*均<0.05)，组间比较显示BHG呈逐渐增加的趋势(组1与组2、3、4，*P*=0.053, 0.086, 0.006)，从组1到4的空腹贡献分别为76.1%、82.6%、81.5%和84.3%。组2、3和4之间的增幅不显著(*P*>0.05)。分析中包括的因素解释了HbA_1C_变异占总体的74%，其中空腹血糖占32.1%的方差，餐后血糖占24.4%的方差。在HbA_1C_>7.3%的情况下，BHG对HbA_1C_的方差贡献更高，达到33.8%。**结论** 本研究提示，相比于餐后高血糖，基础高血糖能更好地预测成人1型糖尿病的整体血糖控制情况。随着HbA_1C_水平的增加，基础高血糖的相对贡献逐渐增加，不同水平的血糖控制在1型糖尿病患者胰岛素调节方面的主要策略也不同。

## 1 INTRODUCTION

Glycemic control is essential in preventing diabetic complications, and it is particularly challenging among patients with type 1 diabetes (T1D) due to the deficiency of beta‐cell function.^1^, ^2^ The glycated hemoglobin (HbA1c), reflecting the overall glycemic exposure for the past 8–12 weeks, was widely accepted as the hallmark measurement of long‐term glucose control while its significant drawback is unable to comprehensively reflect diurnal glycemic fluctuations within days and between days, affected by many factors.^3^, ^4^

Level of HbA1c is acknowledged to be derived from a composite of fasting and mealtime glucose responses.^5^ Since 2003, debates on contribution of postprandial glucose excursions and basal glucose increments to overall hyperglycemia have been pivotally initiated.^6^, ^7^, ^8^, ^9^ However, most of these studies have focused on type 2 diabetes (T2D) particiapnts treated with or without insulin,^10^ or mixed cohorts but composed of only a small percentage of T1D populations,^8^ or analyzed glucose exposure from limited 24‐hour or single glucose value collected via fingerstick blood glucose monitoring (see Supplementary Table S1). Evidence for the exact contribution of daily postprandial glucose exposure in HbA1c in individuals with T1D is few,^11^, ^12^, ^13^ with even less for Asian populations. Considering the biochemical and pathophysiological differences between T1D and T2D, a further discussion in T1D is essential to help improve its clinical management.

In addition to glycemia, HbA1c concentration is also determined by nutrition deficiencies that affect the lifespan of erythrocytes, genetic factors, glycemic variability,^14^ and the other non‐glycemia factors such as age and adiposity potentially.^4^ The emerging continuous glucose monitoring (CGM), advantageously captures daily glucose profiles and separates various components of glycemic exposures at different time periods, providing an opportunity to identify which factors contribute most to long‐term glycemic control more accurately, including those normally experienced, daily postprandial exposures and daytime glucose variability.^15^ Therefore, in the current analysis, based on the CGM data collected in free‐living condition, we first illustrated the relative contribution of basal and postprandial increments above normal levels among T1D adults with different level‐stratification of glycemic control. Moreover, we aimed to compare the strength of associations of real‐life basal and postprandial hyperglycemic exposures at different time periods as well as glycemic variability with HbA1c concentrations and to estimate the variance in HbA1c explained by these factors and the non‐glycemic factors.

## 2 MATERIALS AND METHODS

The present study consisted of the CGM data collected from two clinical trials (NCT 03522870; ChiCTR1900026667). Briefly, the former study is a randomized clinical trial (RCT) and CGM data extrated were those collected during screening period and baseline. The latter study is Guangdong Type 1 diabetes mellitus Translational (GTT) study, an observational cohort study with annual follow‐up visit. Detailed information regarding each study has been previously published.^1^, ^16^ Ethical permissions were both received from ethics commission, and informed consent was obtained from all participants. The studies were conducted in accordance with the Declaration of Helsinki.

### 2.1 Research Design

#### 2.1.1 Patients

Adult patients (age ≥ 18 years) with T1D diagnosed for at least 1 year were included. All patients were on stable treatment with either multiple daily injections or continuous subcutaneous insulin injections. Those with any condition that could affect the reliability of HbA1c measurement (eg, hemoglobinopathy, hemolytic anemia, chronic liver disease) were excluded. The flow chart of the patient selection and data set selection was presented in Figure S1 (see supplementary materials).

#### 2.1.2 Data collection

CGM data collected from the RCT were via professional CGM (Ipro2®, Medtronic) and data collected in GTT study were from patients who were willing to use Ipro2® for at least 1 week during the follow‐ups. Demographic characteristics were both collected by qualified endocrinologists. Before visiting, patients were instructed to have an overnight fast. Fasting venous blood samples were further collected. HbA1c values were tested via the automated analyzer (Bio‐Rad D10; Bio‐Rad Laboratories, Hercules, CA) using the high‐performance liquid chromatography technique. During the entire CGM‐wearing period, fingerstick glucose concentrations were required at least three times per day for sensor calibrations. Patients were encouraged to record daily diet, exercise, insulin adjustment, and the corresponding time in the free‐living settings.

#### 2.1.3 Data Processing

Only the data sets with adequate glucose data (≥70% per day) and the complete three‐meal records per day for at least 48 h were included into analysis. CGM data allowed group means and individual‐event outcomes to be analyzed across 24 h with midnight taken as the start of the nocturnal period of 22:00–06:00 and daytime glucose fluctuations to free‐living interventions defined as 06:00–22:00. Mean fasting blood glucose (FBG), assessing glycemic control at the start of daytime, was calculated from glucose datasets between −30 and 0 min before breakfast. Breakfast period was uniformly set as the period at 06:00–10:00, with the lunchtime set as 11:00–14:00 and dinner time set as 17:00–20:00. The mean preprandial glycemia at each meal was calculated from glucose data sets between −30 and 0 min before meals. Postprandial state was defined as a 3‐h period following ingestion of a meal.

Area under the curve above 110 mg/dL (AUC_>110mg/dL_) in the 24‐h glucose profile was defined as overall hyperglycemia (AUC _total_) and then stratified with postprandial hyperglycemia (PHG, AUC_PHG_ represents for AUC_>110mg/dL_ in the 3‐h period after each meal) and the basal hyperglycemia (BHG, AUC_BHG_ respresents for AUC_>110mg/dL_ in the remaining period). The baseline value of 110 mg/dL was chosen because this threshold has been defined as the upper limit of normal postprandial glucose at fasting or preprandial times by American Diabetes Association and previous studies had also demonstrated that glucose threshold below than 90 mg/dL seemed to be not correlated with HbA1c among T1D populations.^17^ Similar to the previous studies,^6^ the relative contributions of BHG and PHG to overall hyperglycemia were calculated as ((AUC_total_ minus sum of AUC_PHG_ per day)/ AUC_total_) × 100% and (sum of AUC_PHG_/AUC_total_) × 100%, respectively. (Detailed calculation was provided in the Figure S2, see supplementary materials).

Glycemic metrics at different identified time periods were calculated according to the recommendations of the 2017 International Consensus^1^, ^18^: euglycemia: time‐in‐range 70–180 mg/dL (TIR)^2^; hypoglycemia: time‐below‐range (TBR, defined as range < 70 mg/dL or < 54 mg/dL)^3^; hyperglycemia: time‐above‐range (TAR, defined as range > 180 mg/dL or > 250 mg/dL)^4^; glycemic variability: SD, coefficient of variation (CV), mean blood glucose fluctuation (MAGE), and mean of daily differences (MODD).

### 2.2 Statistical analysis

Data were presented as mean ± SD or median [quartiles] according to the distribution. Statistical analysis was performed using R (Version 3.5.2) and SPSS‐27.0 (IBM SPSS Statistics 25, IBM Corporation, USA). The trapezoidal method was used to calculate all AUCs. Participants were grouped according to the quartiles of HbA1c concentrations. Demographics data were compared over groups by using one‐way analysis of variance (ANOVA), followed by a Bonferroni's test. All tests were two sided, and statistical comparisons were considered significant when adjusted *p* values were ≤.05.

When comparing the relative contribution of BHG and PHG, linear regression analysis was used to evaluate the influence of fasting and postprandial hyperglycemia on HbA1c levels, unadjusted and adjusted with age, gender, diabetic duration, treatment, and body mass index (BMI). The relationship between HbA1c and CGM metrics at different time periods was analyzed by Pearson correlation. Comparisons of CGM metrics and relative contribution among groups were also analyzed in the Bonferroni's post hoc test in the linear regression model. Prior to analyses, all CGM‐derived metrics were normalized to facilitate direct comparisons of the strength of their respective associations with HbA1c. In the linear regression model evaluating the proportion of variance in HbA1c explained, CGM‐derived metrics categories such as fasting glycemia (preprandial glucose, AUC_BHG_), postprandial glycemia (AUC_PHG_), glycemic variability (MAGE, MODD, CV, SD), and non‐glycemia factors (age, BMI, diabetic duration, and hemoglobin [Hb]) were estimated. To accommodate for correlations between CGM‐derived metrics, we used their combined contribution to explain variance in HbA1c.^19^

## 3 RESULTS

### 3.1 Participant characteristics

A total of 169 550 glucose data with 2409 meals recorded from 102 patients (male/female, 34/68) were analyzed in this study. The mean age and median diabetes duration were respectively 35.2 ± 12.6 years and 8.9 (2.9, 13.0) years, and the mean BMI was 21.8 ± 3.0 kg/m^2^. There were 51.0% (52/50) of patients receiving insulin pumps. According to the quartiles of HbA1c concentrations, data sets were divided into four groups, with group 1 of HbA1c ≤ 6.7% (*n* = 27), group 2 of HbA1c between 6.7 and 7.3% (*n* = 30), group 3 of HbA1c between 7.3 and 7.8% (*n* = 23), and group 4 of HbA1c ≥7.8% (*n* = 22). The mean HbA1c concentration and the other demographic characteristics among four groups were presented in Table 1. Demographics were similar between included population and those being excluded.

**TABLE 1:** Demographics and overall glycemic controls among patients included into this analysis.

	Total (*N* = 102)	Group 1 (*n* = 27) (A1C ≤ 6.7%)	Group 2 (*n* = 30) (A1C = 6.7–7.3%)	Group 3 (*n* = 23) (A1C = 7.3–7.8%)	Group 4 (*n* = 22) (A1C ≥ 7.8%)	*p*‐value
Sex (F/M)	68/34	18/9	22/8	15/8	13/9	0.779
Age (years)	35.24 (12.59)	31.92 (9.94)	35.16 (13.69)	39.31 (14.50)	35.18 (11.35)	0.238
Duration (years)	8.88 [2.86, 13.00]	4.00 [1.78, 11.05]	9.88 [5.57, 13.33]	5.97 [3.25, 12.99]	10.54 [4.07, 15.33]	0.089
HbA1c (%)	7.25 (0.88)	6.17 (0.51)	7.09 (0.17)	7.60 (0.15)	8.44 (0.42)	<0.001
CSII/MDI (n/n)	52/50	21/6	12/18	12/11	7/15	0.007
BMI (kg/m^2^)	21.75 (3.02)	21.39 (2.63)	21.52 (3.03)	22.93 (3.78)	21.27 (2.40)	0.205
Hb (g/L)	132.52 (14.68)	130.63 (14.23)	132.63 (17.02)	134.30 (15.20)	132.82 (11.65)	0.855
Wearing Days (d)	5.90 (2.97)	5.30 (2.85)	6.93 (3.31)	5.83 (2.66)	5.32 (2.75)	0.008
FBG (mg/dL)	157.19 (28.55)	135.33 (24.47)	152.08 (15.83)	157.81 (21.71)	190.32 (23.16)	<0.001
CGM metrics (Overall)						
MBG (mg/dL)	159.08 (26.48)	132.25 (19.56)	153.54 (13.61)	167.80 (15.00)	190.45 (17.98)	<0.001
eHbA1c (%)	7.17 (0.92)	6.24 (0.68)	6.98 (0.47)	7.47 (0.52)	8.26 (0.63)	<0.001
GMI (%)	7.12 (0.63)	6.47 (0.47)	6.98 (0.33)	7.32 (0.36)	7.87 (0.43)	<0.001
TIR in 70–180 mg/dL (%)	63.04 (15.63)	76.25 (13.53)	67.37 (11.70)	57.15 (9.86)	47.07 (10.05)	<0.001
TAR > 180 mg/dL (%)	31.46 [21.22, 41.80]	16.45 [9.18, 21.80]	30.54 [21.41, 33.46]	37.85 [28.99, 44.29]	49.10 [42.88, 60.12]	<0.001
TAR > 250 mg/dL (%)	8.65 [3.81, 15.16]	2.66 [0.90, 5.09]	7.09 [2.62, 12.75]	12.31 [8.41, 17.73]	17.17 [12.06, 20.77]	<0.001
TBR < 54 mg/dL (%)	0.11 [0.00, 1.46]	0.00 [0.00, 1.56]	0.33 [0.00, 1.67]	0.79 [0.06, 1.85]	0.00 [0.00, 0.00]	0.005
TBR < 70 mg/dL (%)	2.41 [0.52, 6.35]	2.71 [0.61, 13.73]	3.44 [0.95, 5.93]	3.99 [1.76, 6.85]	0.64 [0.00, 1.52]	0.001
CV (%)	32.51 (8.03)	32.14 (9.30)	33.16 (8.26)	35.68 (6.61)	28.74 (6.05)	0.034
SD (mg/dL)	51.36 (14.02)	42.88 (13.44)	50.82 (13.93)	59.17 (10.96)	54.36 (12.58)	<0.001
MAGE (mg/dL)	110.07 (37.88)	85.68 (27.59)	111.99 (43.33)	125.39 (30.76)	121.38 (34.85)	<0.001
MODD (mg/dL)	60.92 (19.25)	47.02 (16.46)	60.97 (18.27)	70.55 (17.63)	67.85 (16.12)	<0.001

*Note*: *Between‐group comparisons were calculated using ANOVA (Bonferroni's post‐hoc test) in the mixed‐effects linear regression, which is fitted with random effects for individuals and fixed effects for HbA1c groups, and adjusted for age, gender, diabetic duration, treatment and body mass index.

Abbreviations: CGM, continuous glucose monitoring; CSII, continuous subcutaneous insulin injection; CV, coefficient of variation; FBG, fasting blood glucose; GMI, glucose management indicator; Hb, hemoglobin; MAGE, mean amplitude of glycemic excursions; MBG, mean blood glucose; MDI, multiple daily injection; MODD, mean of daily differences; SD, standard deviation; TAR, time above range; TBR, time below range; TIR, time in range.

### 3.2 Glycemic control at different time periods among HbA1c groups

**FIGURE. 1:**
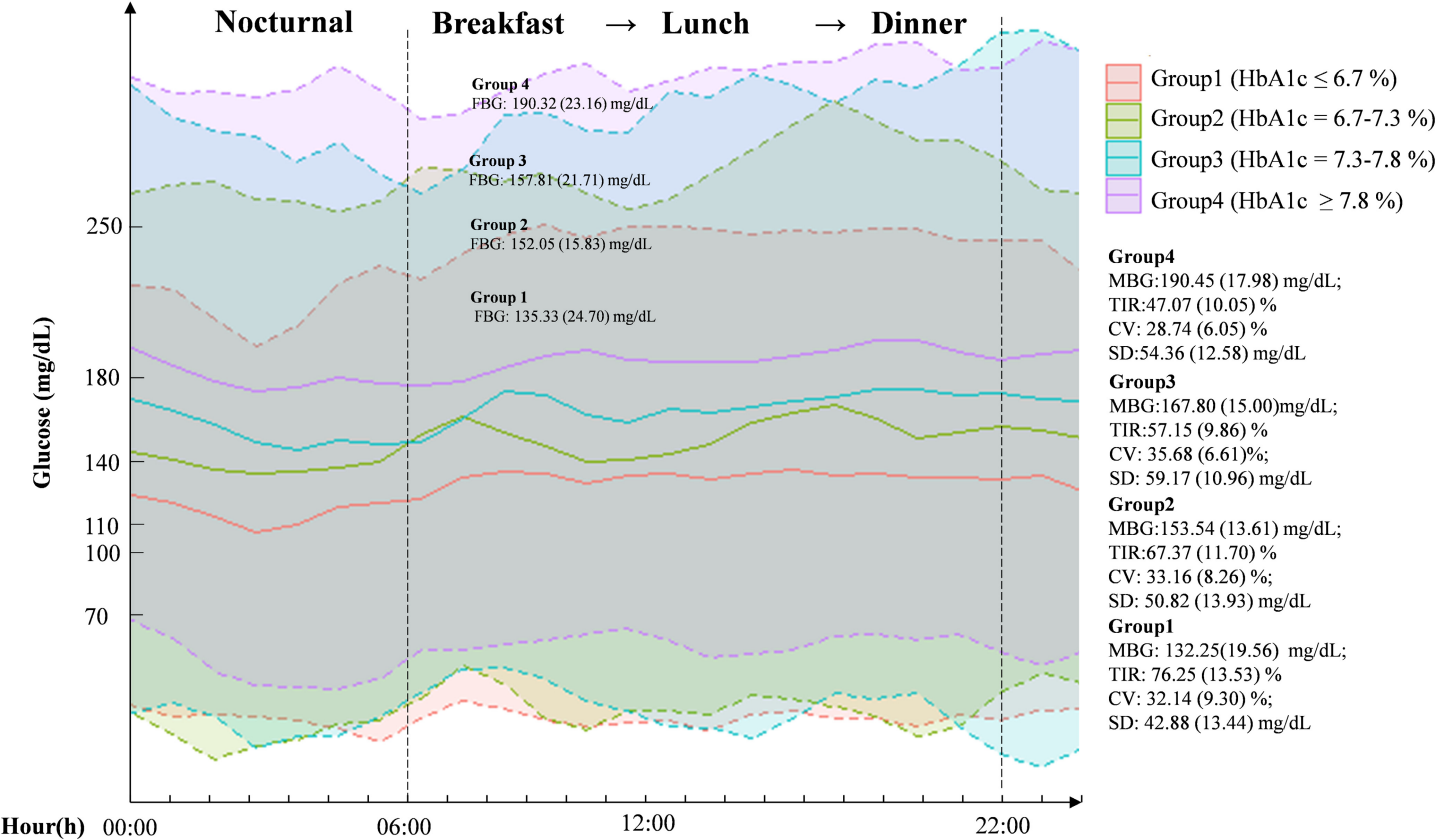
Glucose profiles of the whole 24 h among different HbA1c groups. CV, coefficient of variance; FBG, fasting blood glucose; HbA1c, glycated hemoglobin; MBG, mean blood glucose; TIR, time spent in range 70–180 mg/dL. Solid line represents the mean glucose concentrations at the respective hour; dashed line represents the upper and lower estimates of mean glucose concentrations.

Following removal of ineligible datasets, the median number of days with valid CGM measurements in the analysis was 5.2 (3.0, 7.0) days. Among four HbA1c groups, distinct glycemic patterns within day were presented in Figure 1 and detailed information was in Table 1. Overall, the mean blood glucose, as well as glucose management indicator (GMI), TAR>180 mg/dL, and TAR>250 mg/dL, significantly increased with HbA1c values increasing among groups (*p* < 0.001) and TIR progressively decreasing. Significant differences in mean blood glucose (MBG) and TIR during daytime and nighttime were both robustly observed among groups but there were no significant between‐group differences in CV and SD during nighttime (see Figure 1).

Of note, difference in FBG was not observed between group 2 and group 3 (152.08 ± 15.83 mg/dL vs 157.81 ± 21.71 mg/dL, *p* = .246) whereas group 3 had significantly lower TIR (57.15 ± 9.86% vs 67.37 ± 11.70%; *p* <.001) and higher SD (59.17 ± 10.96 mg/dL vs 50.82 ± 13.93 mg/dL; *p* = .027) and TAR than those in group 2. Furthermore, when assessing CGM metrics during different mealtimes respectively, differences in TIR and TAR>180 mg /dL were observed during breakfast (*p* = .074; *p* = .061) and during lunch (*p* = .036; *p* = .065) rather than those during dinner (*p* = .179; *p* = .202) between group 2 and group 3. TBR < 70 mg/dL and CV between group 2 and group 3 was not observed (*p* both >.05). For other between‐group comparison, TIR during breakfast, lunch, and dinner was significantly lower in group 1 than those in group 3 and 4 whereas difference in TIR tended to be significant only during dinner between group 1 and group 2 (*p* = .069). Detailed information was presented in Figure S3 (see Supplementary Materials).

### 3.3 Relationship between CGM‐derived metrics at different time periods and HbA1c measurements

Association of HbA1c measurements with CGM‐derived metrics including FBG, AUC _total_ (r^2^ = 0.693), MAGE, MODD, and SD were statistically significant (*p* all <.001) except for CV (*p* = .884). When distributing the overall hyperglycemia into basal and postprandial status, both associations were statistically significant with higher r^2^ in AUC _BHG_ of 0.624 (*p* < .001) and AUC_>110mg/dL_ during dinner of 0.412 (*p* < .001).

### 3.4 Contributions of basal and postprandial hyperglycemia and nonglycemic factors to HbA1c variations

**FIGURE 2:**
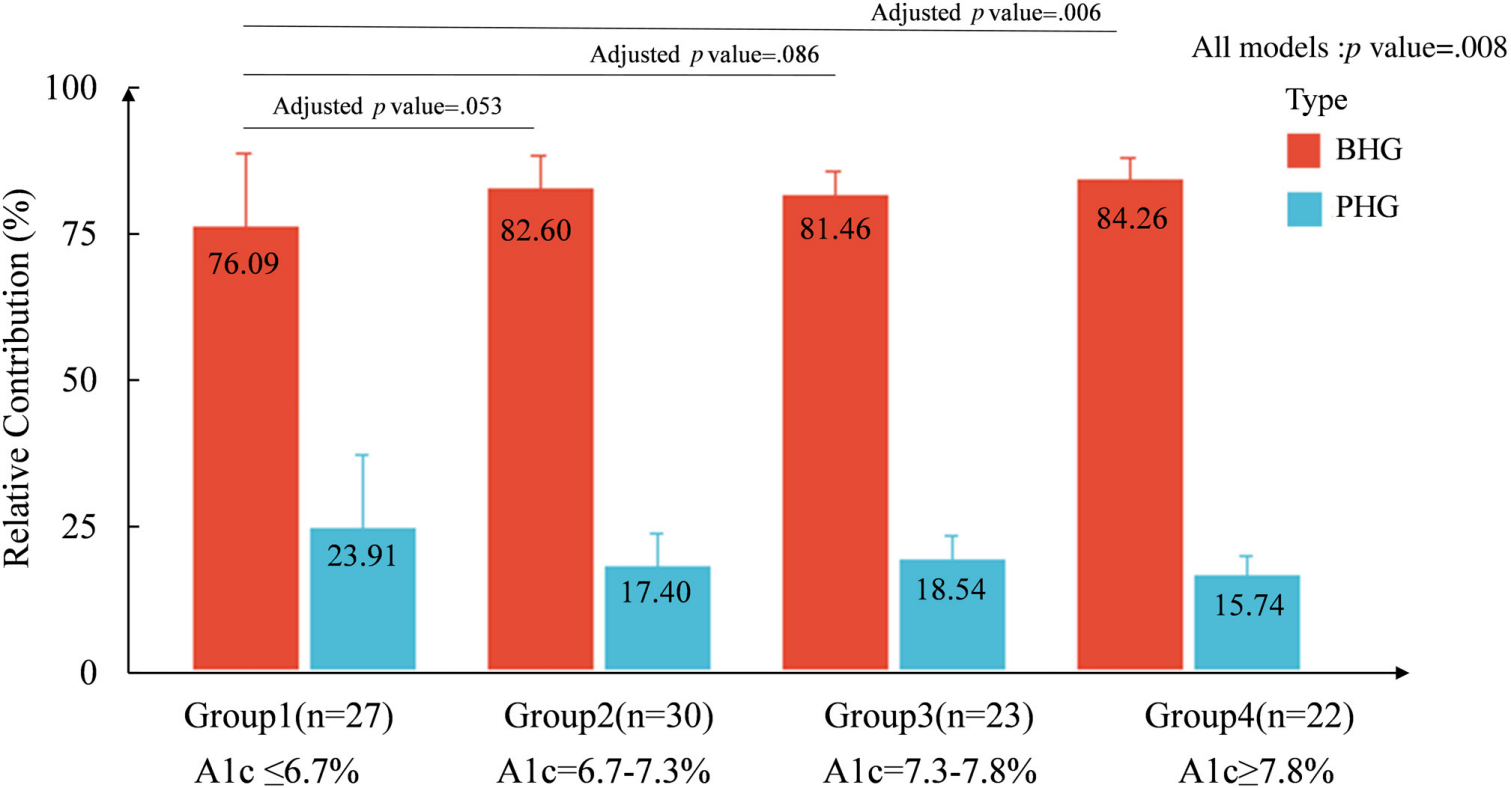
Relative contributions of BHG and PHG to overall hyperglycemia by A1C category. The linear model was adjusted by age, diabetic duration, gender, BMI and type of insulin therapy. BHG, basal hyperglycemia; PHG, postprandial hyperglycemia.

When assessing the relative contribution of BHG and PHG, as presented in Figure 2, the BHG value was approximately four times higher than the PHG value, with fasting contribution of 76.09%, 82.60%, 81.46%, and 84.26% from group 1 to 4 (all *p* < .05). Furthermore, for each relative fasting or postprandial contribution, analyzed individually, comparisons over four groups showed significant differences with the BHG value exhibiting a progressive increase from group 1 to 4 (group 1 vs. 2, 3, and 4, *p* = .053, .086, .006, respectively), but the increase was small with differences in BHG not significant among group 2, 3, and 4. The use of insulin pumps, age, gender, and BMI did not significantly change the relative contribution BHG to HbA1c (*p* all > .05).

**FIGURE 3:**
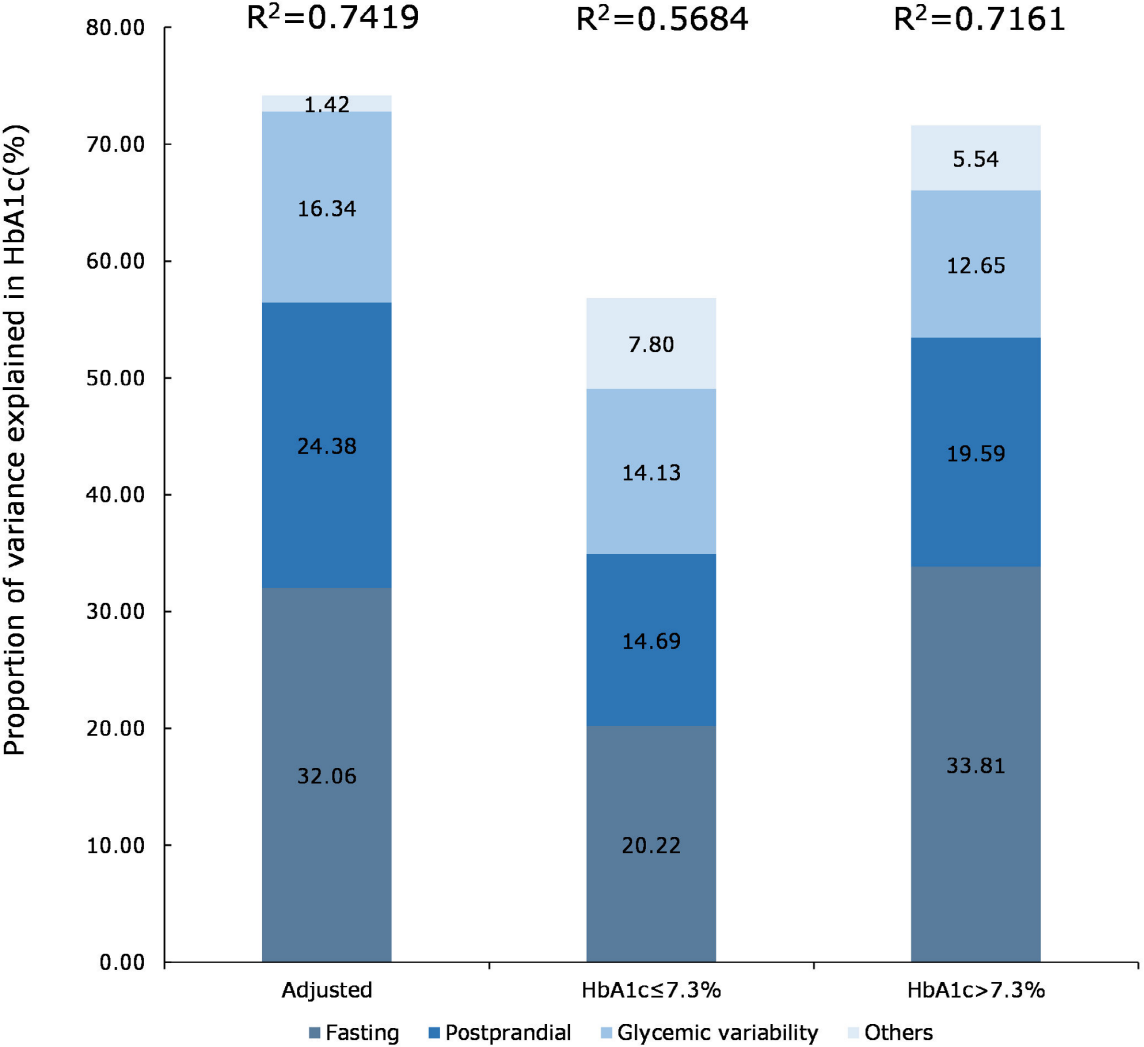
Proportion of variance explained in HbA1c in fasting glycemia (AUC_BHG_ and preprandial glucose), postprandial glycemia (AUC_PHG_), and glycemic variability (MAGE, MODD, CV, SD) adjusted by non‐glycemic factors (age, BMI, Hb, diabetic duration) in all patients (*n* = 102), group with HbA1c <7.3% (*n* = 57), and group with HbA1c ≥7.3% (*n* = 45). AUC_BHG_, area under the curve for fasting hyperglycemia; AUC_PHG_, area under the curve for postprandial hyperglycemia; BMI, body mass index; CV, coefficient of variation; HbA1c, glycated hemoglobin; MAGE, mean amplitude of glycemic excursions; MODD, mean of daily differences.

Proportion of variance in HbA1c explained by fasting glycemia (AUC_BHG_, preprandial glucose), postprandial glycemia, glycemic variability, and non‐glycemic factors is presented in Figure 3. In this study, approximately 74% of the variance of HbA1c was explained by the included factors and the rate was higher among patients with HbA1c >7.3% of 71.6% whereas only 56.8% among patients with HbA1c ≤7.3%. Overall, BHG accounted for a third of the variance explained (32.1%). The proportion was larger in poorer glycemic control (HbA1c >7.3%) with 33.8% than that in HbA1c ≤7.3% group. Further, the proportion explaining the HbA1c variation was progressively smaller in PHG and glycemic variability, with the value of 24.4% and 16.3%. These results were robust in poorer glycemic control whereas the proportion in glycemic variability was similar to that in PHG among patients with HbA1c ≤7.3%. In addition, even after adjusting for the non‐glycemic factors we included, there was still approximately 26% of the variance of HbA1c not explained among all patients. The adjusted and unadjusted mean and 95% confidence interval in HbA1c by each SD difference in CGM metrics was presented in Supplementary Figure S4.

## 4 DISCUSSION

Through the analysis of CGM measurements collected in free‐living settings, we found that BHG better predicts overall glycemic control than PHG or glucose variability exposure to variation in HbA1c among adults with T1D and contributes more among patients with HbA1c >7.3%. We also found that PHG contributed equally but slightly less than glucose variability among patients with HbA1c ≤7.3%.

Since Monnier et al pivotally demonstrated the relative contribution of blood glucose in T2D,^6^, ^7^ the increasing bulk of evidence in T2D had been reported although few had concentrated on T1D, either focusing on adolescents^11^ or only males,^13^ or using self‐monitoring blood glucose (SMBG) data.^12^ Therefore, in our study, focusing on adults with T1D, we found that BHG contributes the majority of diurnal hyperglycemia regardless of levels of HbA1c. This is also consistent with the results observed in the previous studies using the SMBG records.^12^ The higher contribution of BHG among T1D also explains why the emerging advanced closed‐loop automated insulin delivery system, which was currently able to adjust the basal glycemia automatedly, could have outstanding effects in glycemic control with approximately 75%–85% glucose levels maintained in target even though the precise and evidence‐sufficient strategies of postmeal glycemic control have not been fully developed.^20^

Furthermore, our study also observed a different glycemic deterioration pattern among HbA1c groups even though BHG dominates among T1D. As presented, in patients with HbA1c ≥7.8%, BHG dominates and the significantly lower CV value in this group potentially indicated that postprandial excursions might have a relatively small impact. For those with HbA1c <6.7%, even though both of the glycemic targets (HbA1c < 7.0% and TIR≥70%) were achieved, the high risk of hypoglycemia during mealtimes, particularly breakfast, should be of great attention, as higher TBR was observed during this meal depsite non‐signficantly (see Supplementary Figure S3). Of note, even though similar and near‐normal glucose values at the prebreakfast time were observed in group 2 and group 3, patients in group 3 exhibited an elevation of glucose levels from breakfast to lunch, thus leading to less stable glycemic fluctuations and the significantly lower TIR in the following hours. From these results, we deduced that the strategy for insulin titration in T1D patients might follow a three‐step process: (a) for those with higher HbA1c levels, control of the basal diurnal hyperglycemia is predominant; (b) for those with moderate glycemic control (defined as 6.7%–7.8% in our study), the additional concomitant therapy to reduce glycemic fluctuations during meals particularly those in the morning is necessary for further achievement of glycemic targets; and (c) for those with good glycemic control, the treatment target might be the control of postprandial fluctuation, avoiding the hypoglycemic events. However, as our study's sample size was relatively small, whether these deductions are reliable and effective in clinical settings should be further discussed.

Previously, it has been demonstrated that variances existed between CGM metrics and HbA1c and could be determined by many factors such as time period, variation in glycation rates, and factors such as anemia. In this study, the factors derived from CGM data contributed >70% of the variance in HbA1c, which is in line with the recently published data conducted among T1D males from Europe.^13^ Of note, the non‐glycemia factors included in our study, such as age, BMI, diabetic duration, and Hb level, have a small contribution in HbA1c and there was still approximately 20%–30% of the variance in HbA1c undefined even though the contribution of glycemia was higher among those with HbA1c >7.3% . Recently, Ajjan and his colleagues had reported a model that incorporated erythrocyte lifespan, attempting to address limitations in laboratory HbA1c.^21^ Other potential factors that influenced HbA1c level remain an area for future work.

To our knowledge, our study is the first to quantify contribution of different factors to HbA1c among adults with T1D, which made up a large proportion and high‐risk subpopulation of the entire T1D population. The results might have larger generalizability. Furthermore, via application of CGM device, we carefully described glucose profiles in life circumstances without diet controls, and this is also for the first time the variance in HbA1c among Asian populations was estimated. In our study, the number of meals evaluated among T1D patients is large even though detailed information about food intake was still lacking. Through the carefully characterized glycemic excursions occurring after meals described in the free‐living conditions among T1D patients, it might be of great clinical importance for identifying specific periods and individuals with further glycemic management strategies.

A limitation of this study is the lack of detailed information reported to potentially influence postprandial hyperglycemic excursions, such as the nutrient components of the food intake, the behaviors including the timing and dosage of real‐time bolus insulin. As the diet diary was encouraged rather than compulsory, the median durations of the included data sets were only 5 days instead of the whole monitoring 14 days. Besides, the level of beta‐cell function lacked sufficient data collected in two studies. As mentioned previously, the C‐peptide level might attribute to the different contributions of BHG and PHG to HbA1c. Regarding this, further analysis is needed to explain the findings in our study.^22^ Furthermore, the partial data sets reanalyzed in this study were from the screening period and baseline in the RCT instead of the trials initially designed to assess the associations, which might also hamper the generalizability of the results.

## 5 CONCLUSIONS

In conclusion, the present study provides detailed glucose profiles among adult patients with T1D in free‐living settings. It had demonstrated that BHG better predicts overall glycemic control than PHG in T1D and contributes more to glycemic control becoming poorer. Strategies for insulin titration among adult T1D might be adapted according to HbA1c concentration, with predominantly controlling BHG in poorer glycemic control and postprandial glycemia when glycemic control is moderately poor while avoiding hypoglycemia particularly during breakfast to maintain glucose in the normal glucose range.

## AUTHOR CONTRIBUTIONS

Yongwen Zhou and Mao Zheng was the joint first author. Yongwen Zhou, Mao Zheng, and Hongrong Deng contributed to participant recruitment, data collection, and analysis. Yongwen Zhou contributed to manuscript writing. Xueying Zheng, Sihui Luo, Xiaodong Mai, Wen Xu, and Mao Zheng contributed to participant recruitment and data collection. Daizhi Yang contributed to statistical analysis. Jinhua Yan and Jianping Weng designed and supervised the study and contributed to the writing of the manuscript and its final content.

## CONFLICT OF INTEREST STATEMENT

The authors have no conflict of interest to declare.

## Supporting information


**Figure S1.** Flow chart of the dataset selection included in this study.
**Figure S2.** Calculation Methods of AUC.
**Figure S3.** Glycemic control at the three mealtimes in different groups stratified by HbA1c.
**Figure S4.** Mean (95% CI) difference in HbA1c (%) by 1‐SD difference in CGM‐derived glycemic metrics.

**Table S1.** Detailed information for all published studies in this field.

## Data Availability

The data sets used during the current study are available from the corresponding authors on reasonable request.
