# Supplementary material for: Relative contributions of fasting and postprandial glucose increments, glycemic variability, and non‐glycemic factors to HbA1c in individuals with type 1 diabetes
Source: J Diabetes. 2023 May 4;15(6):465–73. doi: 10.1111/1753-0407.13388 (PMC10270743; doi:10.1111/1753-0407.13388)
Supplement: Supplementary file 1 — Figure S1. Flow chart of the dataset selection included in this study. Figure S2. Calculation Methods of AUC. Figure S3. Glycemic control at the three mealtimes in different groups stratified by HbA1c. Figure S4. Mean (95% CI) difference in HbA1c (%) by 1‐SD difference in CGM‐derived glycemic metrics. [file JDB-15-465-s002.pdf]

Supplementary Materials

Figure S1. Flowchart of the dataset selection included in this study

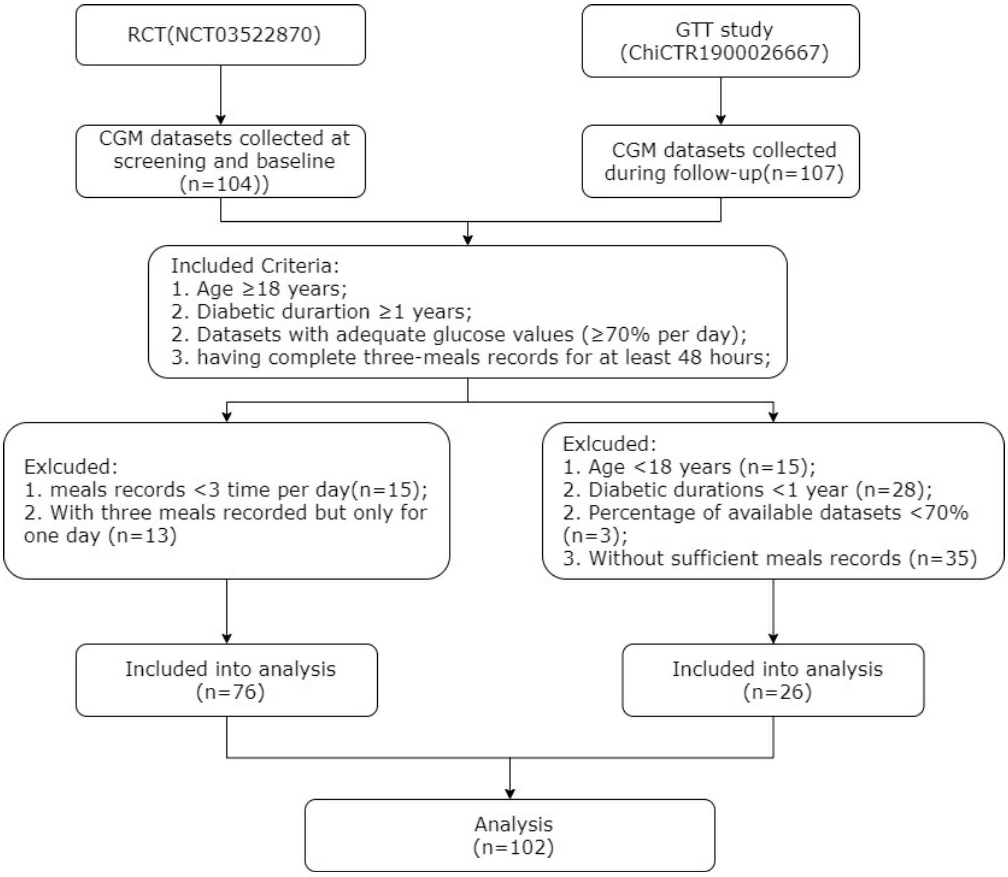

Figure S2 Calculation of the AUC

(A) (B)

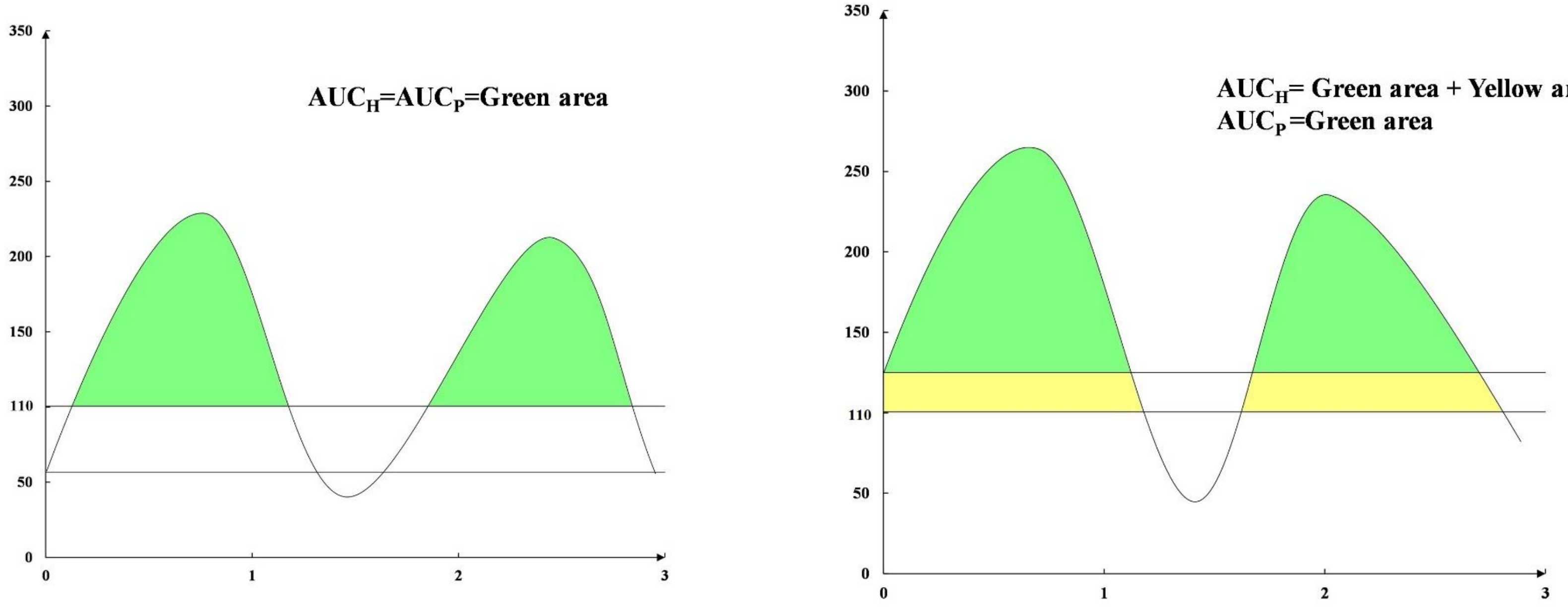

When pre-prandial glucose was under 110mg/dL

When pre-prandial glucose was above/equal 110mg/dL

Fasting hyperglycemia = AUC_H- AUC_P; **Postprandial hyperglycemia** = AUC_P;

Fasting contribution (%) [FHG]= (AUC_H-AUC_P)/AUC_H; **Postprandial contribution (%)[PHG]** = AUC_P/ AUC_H.

Notes: AUC_H was the area above 110 mg/dL; AUC_P was the area above 110 mg/dL and above pre-prandial glucose level;

Figure S3. Glycemic control at the three mealtimes in different groups stratified by HbA1c

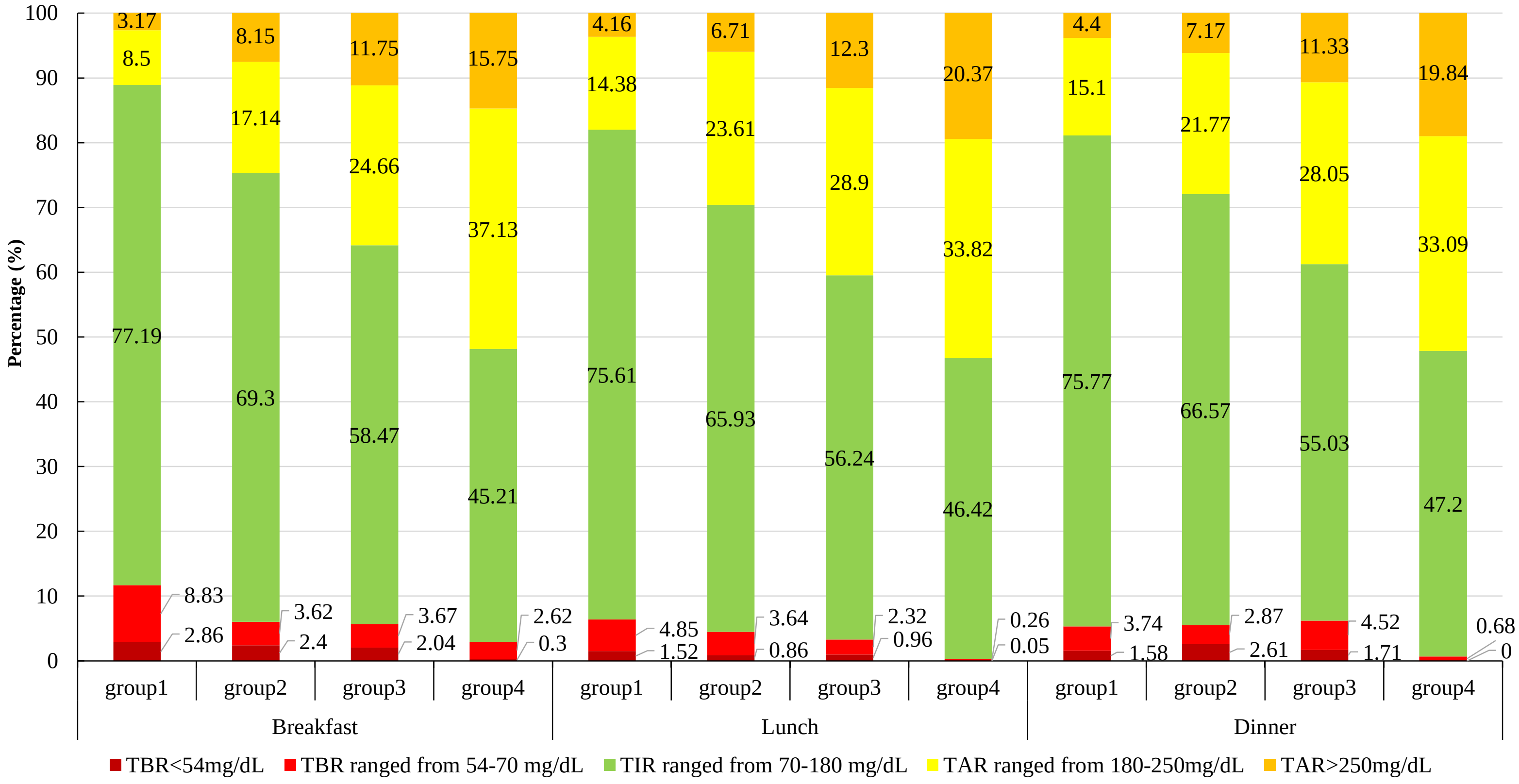

Figure legends: Notes: TBR, time below range; TIR, time spent in range; TAR, time above range.

Figure S4. Mean (95%CI) difference in HbA1c (%) by 1-SD difference in CGM-derived glycemic metrics.

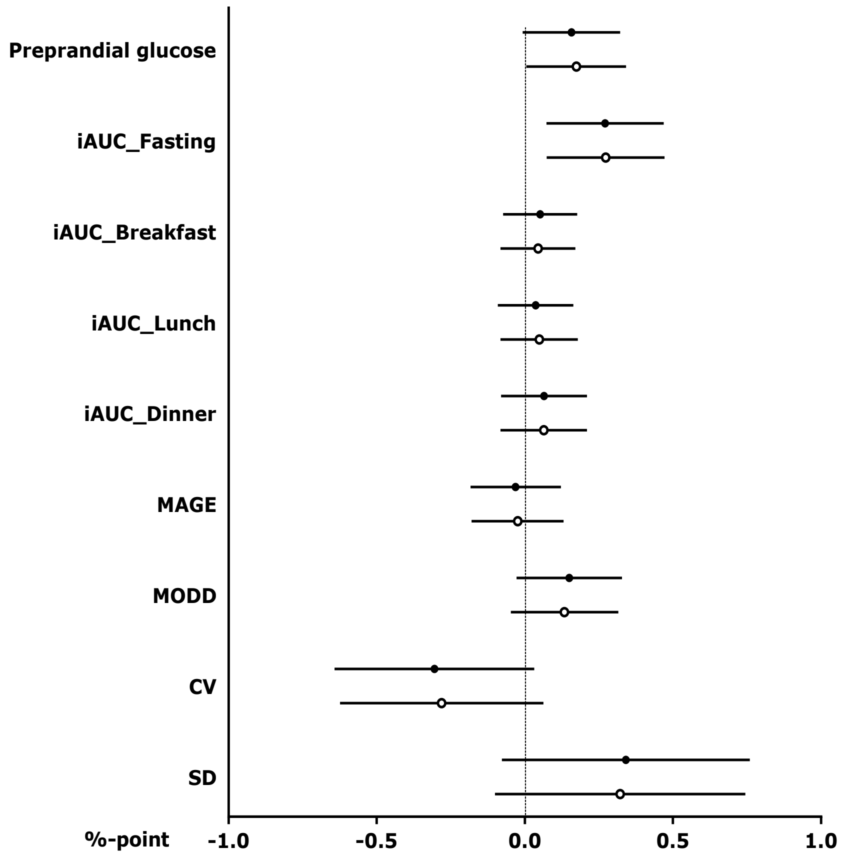

Figure legneds: Estimated differences are unadjusted (dotted) and adjusted (dashed) for age, body mass index and Hb.
