# Supplementary material for: Relative contributions of fasting and postprandial glucose increments, glycemic variability, and non‐glycemic factors to HbA1c in individuals with type 1 diabetes
Source: J Diabetes. 2023 May 4;15(6):465–73. doi: 10.1111/1753-0407.13388 (PMC10270743; doi:10.1111/1753-0407.13388)
Supplement: Supplementary file 2 — Table S1. Detailed information for all published studies in this field. [file JDB-15-465-s001.docx]

| **Table S1. Detailed information for all published studies in this field** | | | | | | | | | | |
| --- | --- | --- | --- | --- | --- | --- | --- | --- | --- | --- |
| **Study** | **N** | **Types of patients** | **Treatment** | **Ethnity** | **Hyperglycemia Threshold (mg/dL)** | **Glucose data** | **Diets** | **HbA1c (%)** | **Groups of HbA1c** | **Relative of PPG** |
| Monnier et al. (2003)(1) | 290 | T2DM on diet, SU or MTF | Non-insulin & non-AGI treated | Caucasians | ≥110 | Four-point SMBG | Two standardized meals | 6.3–11.4 | <7.3%;  7.3-8.4%；  8.4-9.2%；  9.3-10.2%；  >10.2% | From the lowest (69.7%) to the highest quintile of HbA1c (30.5%). |
| N. Hillman et al  (2004)(2) | 71 | T1D | insulin | Spain | Not determined | SMBG | Before three meals | not mentioned | <7.0%;  ≥7.0% | Preprandial glycaemia better predicts overall glycaemic control in Type 1 diabetes than postprandial glycaemia. |
| Woerle et al. (2007) (3) | 164 | Type 2 diabetes on diet control, MTF, SU, NPH, and others | | Caucasians | ≥100 | Seven-point SMBG | Three carbohydrate containing meals | 8.7 ± 0.1 | ≤7% >7.0% | The relative contributions of PPG accounted for nearly 90% when HbA1c < 6.2% but only about 40% when HbA1c > 8.9%. |
| Shimizu et al. (2008)(4) | 57 | 15 T1D  42 T2D | Premix or basal bolus Insulin treated | Japanese | Not dertermined | Six-point SMBG | / |  |  | ND (PPH had better correlation with HbA1c) |
| Peter et al.  (2009) (5) | 52 | T2DM on gliclazide, MET or both | Insulin or acarbose treated | Caucasians | ≥100 | Frequently sampled blood tests | Standard 500-kcal mixed meal (58% carbohydrate, 22% fat and 20% protein) | 7.7 ± 1.0 | <7.3%; 7.3%-8.0%; >8.0% | The relative contributions of PPG accounted for nearly 85.8% when HbA1c < 7.3% but only about 48.6% when HbA1c >8.0% |
| Kikuchi et al. (2010)(6) | 66 | Type 2 diabetes on diet control, MTF/ PIO/SU or basal insulin | Not on prandial or premix insulin & non-AGI treated | Japanese | Not determined | Sixpoint SMBG | Three standardized meals | 5.7–12.5 | <8% ≥8.0% | Postprandial hyperglycemia was strongly correlated with the HbA1c in the group with HbA1c levels less than 8.0% |
| Haddadinezhad S et al  (2010) (7) | 300 | 63 T1D  237 T2D | 53 Insulin | Indian | Not determined | Plasma glucose | Preprandial and postprandial | Not limited | <7.5%  7.5-9.0%  >9.0% | The postprandial (after breakfast) plasma glucose has closer association to glycosylated hemoglobin than fasting plasma glucose. |
| Riddle et al. (2011) (8) | 1699 | T2DM on MTF/ SU or both | Add on basal or premix insulin | 94.6% Caucasians | ≥100 | Seven-point SMBG | / | 8.7  (7.6–10.0) | By 0.5% | The relative contributions of BG played a major role before intensive treatment. |
| Wang et al.  (2011) (9) | 121 | Type 2 diabetes on MTF and SU/AGI | Non-insulin treated | Chinese | ≥100 | 3-day CGM | Three main recorded meals | 5.7–12.7 | <7.1； 7.1-7.5； 7.5-8.0； 8.1-8.7； >8.7 | from 71% to 44% as the increased of HbA1c, up to 70% when HbA1c < 7.1%, about 50% each when HbA1c > 7.6% |
| Peter et al.  (2013) (10) | 52 | Type 2 diabetes on SU/MTF, or both | Non-insulin treated | Caucasians | ≥100 | Multiple venous blood for 4 h after each meal | Three standardized meals | 5.9–9.6 | <7.0 7.0-<7.5 7.5-<8.0 8.0-<9.0 ≥9.0 | The relative contribution of PPG exposure decreased across the groups from 43.5% (HbA1c<7.0%) to 17.8% (HbA1c≥9.0%), whilst the contributions of fasting hyperglycaemia increased from 56.5% to 82.2% |
| Fysekidis et al.  (2014) (11) | 70 | Obesity, BMI > 25 kg/m2 (35.2 ± 6.8 kg/m2) | Without known glycemic disorder | Caucasians | ≥99 | 3-day CGM | Three standardized meals | 5.9 (5.1–7.4) | 5.1-5.5 5.6-5.9 6.0-6.2 6.3-7.4 | The relative contributions of PPG accounted for nearly 81.2% when HbA1c < 5.3% but only about 57% when HbA1c > 6.6% in overweight/obese patients. |
| Kang et al.  (2015) (12) | 59 | Newly diagnosed Type 2 diabetes | Drug-negative | Chinese | ≥110 and NGT curve | 3-day CGM | Three standardized meals | 8.0 (5.6–13.2) | ≤7.0 7.0≤9.0 >9.0 | 77.2% (HbA1c ≤ 7.0%), 55.4%(7%<HbA1c ≤ 9.0%), 22.8% (HbA1c > 9.0%). Overestimated for 10-20% choosing 6.1 mmol/L as baseline compared with NGT curve. |
| Wang et al. (2017) (13) | 229 | Type 2 diabetes on all treatment regimes | 37.7% with OAD and 62.3% with insulin | Chinese | ≥100 | 3-day CGM | Not determined | 8.4 ± 2.1 |  | The relative contributions of PPG accounted for nearly 62.4% when HbA1c < 6.5% but only about 35.8% when HbA1c >8.0% |
| Lim et al.  (2017) (14) | 100 | Type 2 diabetes on all treatment regimes | 58% with insulin | Multi-ethnic (Malays, Indians, Chinese) | ≥100 | 6-day CGM | All recorded meals | 6.0–14.0 | 按1.0划分 | From 49% (HbA1c < 7.0%) to 39% (HbA1c ≥ 10.0%), greater PG contribution when HbA1c was <8%. BG predominated when HbA1c was ≥9and ≥ 10% in oral ntidiabetic drug- and insulin-treated patients |
| Li et al.  (2018) (15) | 81 | T2DM treated with insulin | LM25 and LM50 | Chinese | ≥110 | 4-point blood glucose profile at least one day | high-carbohydrate test meals on day 1 and high-fat test meals on day 2 and their habitual diets on day 3 | 7.8 ± 1.4 |  | PPG played a major role of patients with HbA1c < 8.5%. PPG’s contribution rate decreased with increasing HbA1c (from 65.5% to 39.2%) |
| Reznik et al. (2018)(16) | 259 | T2DM treated by insulin | Non-oral drug treated | Multicenter study (Canada, Europe, United States, Africa) | ≥100 | OPT2mise trial | maintained efforts at lifestyle and dietary management, but carbohydrate counting was not required | 8.9 ± 0.8 | <8 8-8.4 8.5-8.9 9-9.4 ≥9.5% | PPG accounts for only 20% to 30% of overall hyperglycaemia, regardless of the baseline HbA1c level who fail to respond to an intensified MDI regimen. |
| Umpierrez et al.  (2019)(17) | 673 | Type 2 diabetes on dulaglutide | Not determined | Caucasians | ≥100 | Sevenpoint SMBG | Not determined | 8.1 ± 0.94 |  | The relative contributions of PPG accounted for nearly 52% when HbA1c < 7.0% but only about 23% when HbA1c ≥ 9% |
| Yan et al.  (2019) (18) | 305 | Newly diagnosed type 2 diabetes or IGT/ IFG | Drug-native | Chinese | ≥110 | 3-day CGM | Personalized instructions and meals of a total daily caloric intake of 25 kcal/kg/day | 9.3 ± 1.9 | 4.9-6.0 6.1-7.8 7.9-8.7 8.8-9.5 11.0-14.6 9.6-10.9 | Regression analyses indicate that the contributions of FG and PG were equal (both 50%) when the level of HbA1c was 8.5%. FG contributed significantly more than PG in the higher groups of HbA1c (9.6-10.9% and 11.0-14.6%) |
| Moon et al.  (2020) (19) | 194 | Type 2 diabetes on diet control, MTF,SU or DPP4i | Non-insulin & non-AGI treated | Korean | ≥100 | 7-point SMBG | Not determined | 7.0 ± 0.9 | ≤6.6 6.7%-7.1% ≥7.2% | The relative contribution of PPG decreased (55.3%±5.5%, 42.0%±4.4%, 33.5%±2.8%) with the elevated HbA1c (≤6.6%,6.7%-7.1%, ≥7.2%). HbA1c, waist circumference, and triglyceride had a significant association with AUCFHG. Only HbA1c and age was associated with AUCPPG. |
| J. Ma,et al  (2021) (20) | 490 | Normal glucose tolerance；  impaired glucose tolerance  newly-diagnosed T2D  drug-treated T2D | Not determined | Chinese | ≥100 ≥110 NGT CURVE | 24-h profile | Not determined | 6.96 ± 1.26 |  | The 24-h glucose curve of NGT is more suitable for studying the relative contributions of BG and PPG to HbA1c, as it considers physiological fluctuations in NGT after meals. However, 5.6 mmol/L can be used when the 24-h glucose curve for NGT is unavailable; using 6.1 mmol/L as a baseline value may overestimate the contribution to the HbA1c. |
| M. D. Campbell et al  (2022)(21) | 32 | T1D males | Insulin | Europe | Not determined | 4-week CFM | Not determined | 7.3±0.9% | Not determined | Postprandial glucose, specifically evening-time postprandial glucose, is the single largest contributing factor to HbA1c in T1D. |

**Reference**

1. Monnier L, Lapinski H, Colette C. Contributions of fasting and postprandial plasma glucose increments to the overall diurnal hyperglycemia of type 2 diabetic patients: variations with increasing levels of HbA(1c). Diabetes care. 2003;26(3):881-5.

2. Hillman N, Herranz L, Grande C, Vaquero PM, Pallardo LF. What is the relative contribution of blood glucose levels at different time points of the day to HbA1c in Type 1 diabetes? Diabetic medicine : a journal of the British Diabetic Association. 2004;21(5):468-70.

3. Woerle HJ, Neumann C, Zschau S, Tenner S, Irsigler A, Schirra J, et al. Impact of fasting and postprandial glycemia on overall glycemic control in type 2 diabetes Importance of postprandial glycemia to achieve target HbA1c levels. Diabetes research and clinical practice. 2007;77(2):280-5.

4. Shimizu H, Uehara Y, Okada S, Mori M. Contribution of Fasting and Postprandial Hyperglycemia to Hemoglobin A1c in Insulin-Treated Japanese Diabetic Patients. Endocrine journal. 2008;55(4):753-6.

5. Peter R, Dunseath G, Luzio SD, Chudleigh R, Choudhury SR, Owens DR. Relative and absolute contributions of postprandial and fasting plasma glucose to daytime hyperglycaemia and HbA(1c) in subjects with Type 2 diabetes. Diabetic medicine : a journal of the British Diabetic Association. 2009;26(10):974-80.

6. Kikuchi K, Nezu U, Shirakawa J, Sato K, Togashi Y, Kikuchi T, et al. Correlations of Fasting and Postprandial Blood Glucose Increments to the Overall Diurnal Hyperglycemic Status in Type 2 Diabetic Patients: Variations with Levels of HbA1c. Endocrine journal. 2010;57(3):259-66.

7. Haddadinezhad S, Ghazaleh N. Relation of fasting and postprandial and plasma glucose with hemoglobinA1c in diabetics. International journal of diabetes in developing countries. 2010;30(1):8.

8. Riddle M, Umpierrez G, Digenio A, Zhou R, Rosenstock J. Contributions of Basal and Postprandial Hyperglycemia Over a Wide Range of A1C Levels Before and After Treatment Intensification in Type 2 Diabetes. Diabetes care. 2011;34(12):2508-14.

9. Wang JS, Tu ST, Lee IT, Lin SD, Lin SY, Su SL, et al. Contribution of postprandial glucose to excess hyperglycaemia in Asian type 2 diabetic patients using continuous glucose monitoring. Diabetes/metabolism research and reviews. 2011;27(1):79-84.

10. Peter R, Dunseath G, Luzio SD, Owens DR. Estimates of the relative and absolute diurnal contributions of fasting and post-prandial plasma glucose over a range of hyperglycaemia in type 2 diabetes. Diabetes Metab. 2013;39(4):337-42.

11. Fysekidis M, Cosson E, Banu I, Duteil R, Cyrille C, Valensi P. Increased glycemic variability and decrease of the postprandial glucose contribution to HbA1c in obese subjects across the glycemic continuum from normal glycemia to first time diagnosed diabetes. Metabolism: clinical and experimental. 2014;63(12):1553-61.

12. Kang X, Wang C, Chen D, Lv L, Liu G, Xiao J, et al. Contributions of Basal Glucose and Postprandial Glucose Concentrations to Hemoglobin A1c in the Newly Diagnosed Patients with Type 2 Diabetes—The Preliminary Study. Diabetes technology & therapeutics. 2015;17(7):445-8.

13. Wang X, Wang F, Wang H, Li N. Correlation of Fasting Versus Postprandial Plasma Glucose with HbA1c in Chinese Type 2 Diabetic Patients Taking Different Hypoglycemic Agents. Clinical laboratory. 2017;63(7):1293-300.

14. Lim LL, Brnabic AJ, Chan SP, Ibrahim L, Paramasivam SS, Ratnasingam J, et al. Relationship of glycated hemoglobin, and fasting and postprandial hyperglycemia in type 2 diabetes mellitus patients in Malaysia. Journal of diabetes investigation. 2017;8(4):453-61.

15. Li W, Ping F, Xu L, Zhang H, Dong Y, Li H, et al. Contribution of BHG and PPHG to Overall Hyperglycemia in T2DM Patients Treated with LM25 and LM50: Post Hoc Analysis of a Randomized Crossover Trial. Diabetes therapy : research, treatment and education of diabetes and related disorders. 2018;9(4):1605-14.

16. Reznik Y, Habteab A, Castaneda J, Shin J, Joubert M. Contribution of basal and postprandial hyperglycaemia in type 2 diabetes patients treated by an intensified insulin regimen: Impact of pump therapy in the OPT2mise trial. Diabetes, obesity & metabolism. 2018;20(10):2435-41.

17. Umpierrez G, Pantalone KM, Atisso CM, Landó LF, Patel H. Relative contribution of basal and postprandial hyperglycaemia stratified by HbA1c categories before and after treatment intensification with dulaglutide. Diabetes, obesity & metabolism. 2019;21(6):1365-72.

18. Yan R, Hu Y, Li F, Jiang L, Xu X, Wang J, et al. Contributions of Fasting and Postprandial Glucose Concentrations to Haemoglobin A1c in Drug-Naïve Mal-Glucose Metabolism in Chinese Population Using Continuous Glucose Monitoring System. International journal of endocrinology. 2019;2019:1-5.

19. Moon J, Kim JY, Yoo S, Koh G. Fasting and Postprandial Hyperglycemia: Their Predictors and Contributions to Overall Hyperglycemia in Korean Patients with Type 2 Diabetes. Endocrinology and metabolism (Seoul, Korea). 2020;35(2):290-7.

20. Ma J, He H, Yang X, Chen D, Tan C, Zhong L, et al. A new approach for investigating the relative contribution of basal glucose and postprandial glucose to HbA1C. Nutrition & diabetes. 2021;11(1).

21. Campbell MD, West DJ, O'Mahoney LL, Pearson S, Kietsiriroje N, Holmes M, et al. The relative contribution of diurnal and nocturnal glucose exposures to HbA1c in type 1 diabetes males: a pooled analysis. Journal of diabetes and metabolic disorders. 2022;21(1):573-81.
